# Negative experiences with primary care services in Norway expressed in patient and next-of-kin complaints – a qualitative study

**DOI:** 10.1186/s12913-025-12231-9

**Published:** 2025-01-17

**Authors:** Alison Axisa Eriksen, Terje Emil Fredwall, Inger Beate Larsen

**Affiliations:** 1 Centre for Care Research, Faculty of Health and Sports Sciences, University of Agder, Grimstad, Norway; 2 Department of Psychosocial Health, Faculty of Health and Sports Sciences, University of Agder, Grimstad, Norway

**Keywords:** Patient complaints, Complainants, Ombudsman, Primary care services, Next of kin role, Next-of-kin burden, Quality of care

## Abstract

**Background:**

Primary health care has been central to achieving universal health coverage. In Norway, there has been increased pressure on primary care services in recent years. Patient complaints offer key insights into care quality, and qualitative analysis of patient complaints can help healthcare professionals reflect on and improve their practices. The aim of this study is to provide an understanding of negative experiences with primary care in Norway, as expressed in complaints to the Health and Social Services Ombudsman (Ombudsman).

**Methods:**

An explorative descriptive qualitative design was employed. Document analysis was used to examine earlier complaints. A total of 221 complaints were analysed via reflexive thematic analysis. The participants consisted of a sample of patients and next of kin who made complaints regarding primary care services to the Ombudsman in Norway in 2019.

**Results:**

Four themes were developed through thematic analysis: 1) the services patients received did not align with their perceived needs; 2) patients experienced disrupted transitions between healthcare services; 3) patients and next of kin encountered substandard case handling; and 4) insufficient services placed a heavy burden on next of kin. These findings were integrated to a patient-centred framework to provide structure and make them more accessible to healthcare providers.

**Conclusions:**

This study highlights the challenges faced by patients and their next of kin related to Norwegian primary care services, pointing to a gap between the expected quality of healthcare services and the services received and to nudging next of kin to provide informal care.

**Supplementary Information:**

The online version contains supplementary material available at 10.1186/s12913-025-12231-9.

## Background

Since the 1978 Alma-Ata declaration, primary health care has been widely regarded as the most effective means of delivering cost-efficient, essential healthcare services that are critical to achieving universal health care coverage [1]. In Norway, primary health care is regulated by the Municipal Health and Care Act (2011), according to which all citizens have the right to necessary healthcare in the municipality in which they reside [2]. Healthcare in Norway is based on the principles of solidarity and universal coverage [3], with public sources funding 85% of the expenditure [2]. However, governance changes (e.g., new public management) have impacted service quality [3]. In 2012, substantial transformations in primary health care services transpired with the introduction of the Care Coordination Reform [2]. This initiative was designed to transfer the medical and care responsibilities of individuals who do not require specialized care from hospitals to primary care [2].

The Care Coordination Reform resulted in improvements in both horizontal and vertical coordination of public health, enhanced primary care, increased patient choice, and education of healthcare professionals to better meet future healthcare needs [2]. However, this reform has also placed greater responsibility on municipalities to provide 24-hour healthcare services following hospital discharge [2]. Coupled with the fact that people are living longer, this has heightened the demand for specific [2] and more complex primary care services [4]. These challenges, along with potential solutions through the reorganization of service delivery, were explored in a 2015 white paper titled *The Primary Health and Care Services of Tomorrow* Norwegian Ministry of Health and Care Services [4]. Given that demand for services often exceeds supply, primary care settings face challenging prioritization decisions [5].

Healthcare policies in Norway aim to empower patients by facilitating patient-centred care and through extensive patient rights [2, 6, 7]. Patient-centred care is described as a practical framework that fosters healthful relationships between healthcare providers, patients and next of kin [8]. Patient-centred care is founded on the principles of respecting individuals, honouring their right to self-determination and fostering mutual respect and understanding [8]. Patient rights give individuals who have negative experiences with healthcare services the opportunity to contact the Health and Social Services Ombudsman (Ombudsman) and ask for advice and guidance on how to proceed with their inquiry [6].

Challenges in primary care necessitate a better understanding of how they influence the quality of primary care services. This, especially since a review of healthcare services in Norway, states that the quality of primary care services is not regularly measured [2]. Research has shown that patient perceptions and experiences of care can identify critical factors affecting care quality, particularly in relation to patient safety in primary care [9]. Additionally, understanding the performance of primary care services in countries that invest heavily in primary care can contribute to international ongoing discussions on the sustainability and effectiveness of primary care models [1].

Data from patient complaints have been recognized as a valuable tool for revealing failures of healthcare delivery that may not be captured by traditional healthcare assessments [10]. While research has shown that systematic analysis of complaint data via a taxonomy is useful for identifying problem areas in care [11], understanding their complexities may be challenging when these same quantitative methods are used [12]. On the other hand, qualitative studies of patient complaints provide deeper insights into negative experiences with healthcare services, which are more appropriate when deeper meanings of the patient experience are of interest [13].

Simultaneously, patient narratives can inspire healthcare professionals to reflect on their practices [14]. When such experiences are shared and received by others, they have the power to change the recipients [15]. This transformation occurs as the narratives compel the recipients to envision the situation, recollect similar experiences, and establish connections or distinctions with the characters in the narrative [15]. Therefore, qualitative studies on negative patient experiences expressed in complaints could help healthcare professionals and students reflect on their practices. Previous research [13] has shown that negative patient experiences across various healthcare contexts are often comparable, with patients experiencing issues such as limited access to care, lack of continuity, insufficient information and inappropriate care. Therefore, negative experiences with primary care services in Norway may provide valuable insights for other countries.

The aim of this study is to provide an understanding of negative experiences with primary care in Norway, as expressed in complaints to the Ombudsman, to 1) foster a discussion on the perceived quality and prioritization of primary care services and 2) provide healthcare professionals and students with valuable learning opportunities.

## Methods

### Design

In this study, we used an explorative descriptive qualitative (EDQ) design, which is appropriate for investigating and describing participant experiences [16]. The Consolidated Criteria for Reporting Qualitative Studies (COREQ) guidelines [17] were followed to ensure that our study report was comprehensive.

To provide an understanding of negative experiences with primary care, we chose to conduct a document analysis of complaints made by patients or next of kin to the Ombudsman. Document analysis can serve as a standalone method in research, particularly when investigating past events that can no longer be directly observed or recalled accurately by individuals who experienced them [18].

### Setting

#### Norwegian primary care

In Norway, municipalities provide a wide range of primary care services. These include general practitioner (GP) care, out-of-office-hours care at emergency medical centres (EMCs), rehabilitation, maternity and child healthcare, long-term care, nonhospital emergency care beds, services for substance abuse and mental care, and support for informal caregivers [2]. Additionally, municipalities provide user-controlled personal assistance (UPA) to individuals with complex healthcare needs [19].

### Sample

#### Norwegian Complaint System

Individuals with negative experiences with healthcare services in Norway can contact the Ombudsman for information, advice, and guidance [2]. Ombudsman services are regulated by the Patient and User Rights Act and must be provided in every county [6]. The Ombudsman assists with patient rights, helps clarify and submit inquiries, guides through complaints or appeals, accompanies patients to meetings and advises on compensation claims [20].

Individuals who are dissatisfied with healthcare can file a complaint with the service provider [2]. If unresolved, the complaint is sent to the County Governor, which can investigate but cannot award compensation [2]. For compensation, claims must be submitted to the Norwegian System for Patient Injury Compensation [2]. The Ombudsman supports individuals throughout the complaint process and holds relevant documents for each phase.

#### Sample

Our sample is from 2019, during which the Ombudsman documented approximately 4500 complaints regarding primary care services [21].

To make informed decisions about the sample, we gathered information from Ombudsman employees on their interaction with patients or next of kin, documentation practices and the types of complaint records in their system. The majority of patients and next of kin contact the Ombudsman by phone or in-person visits, although some prefer written communication through letters or emails. Verbal complaints are recorded in notes by Ombudsman employees, who, while following no formal guidelines, aim to keep these records objective and focus solely on patients’ issues with healthcare services.

Following advice from Ombudsman employees, some patients choose to send a complaint letter to the healthcare provider. While patients or their next of kin are encouraged to draft these letters themselves, some request assistance from Ombudsman employees, who then write the letter and have the patient or family review and confirm its accuracy before sending.

Initially, we considered analysing only complaints written by patients or next of kin, as these provide subjective knowledge about the experience. We recognized, however, that excluding Ombudsman-written notes and letters could omit the perspectives of individuals who preferred to contact the Ombudsman directly instead of writing their own complaints, potentially creating a gap in understanding negative experiences with healthcare. To avoid this “qualitative fallacy”, where parts of the story are lost when they don’t fit neatly with other data, as described by Bøe, Berthelsen, Larsen, Topor [22], we chose to include all types of complaint records. However, a large sample was selected to allow broader insights to primary care in Norway.

Our final sample included a diverse array of documents, including letters or emails written by patients or next-of-kin and notes documented by Ombudsman employees. The Ombudsman kept records of complaint letters to healthcare providers, the County Governor, and applications to the Norwegian System of Patient Injury Compensation. To embrace a comprehensive range of perspectives, we included all document types, even if some cases were documented by multiple records and others by just one.

Apart from applications submitted to the Norwegian System of Patient Injury Compensation, documents from patients and next of kin were written as free text. Documents varied in length, from brief sentences to detailed multipage accounts of their negative experiences. Notes written by Ombudsman employees also ranged in detail and included dates to track contact and case progression.

#### Data collection

Owing to ethical and legal considerations, an Ombudsman employee extracted a sample of 200 cases, each originating from the 15th of every month (or the nearest working day). Only 18 of these included documents from patients or next of kin; the rest were recorded by Ombudsman employees. To ensure that patient and next-of-kin perspectives were represented, an Ombudsman employee extracted an additional random sample of 50 cases that had documents written by patients or next-of-kin.

The Ombudsman employee extracted data following these criteria:

Inclusion criteria:

1. Documents describing issues individuals of any age had with primary care or with both primary and specialized healthcare services
2. Documents written by patients, next of kin or Ombudsman employees.

Exclusion criteria:

1. Documents describing issues solely related to specialized healthcare services.

After the review of the 250 cases, cases involving prior complaints to the County Governor or unrelated to primary care were excluded. Table 1 summarizes the sample.

**Table 1:** Overview of sample of complaints included in the study

**Documents included in study sample**	**Cases uploaded by Ombudsman employee**	**Cases included in the study**
Notes written by Ombudsman employee	173	152
Complaint written by patients or next of kin	8	7
Notes written by Ombudsman employee + Complaint written by patient or next of kin	44	38
Notes written by Ombudsman employee + Letter written by Ombudsman employee to a healthcare organization or to the County Governor	23	22
Notes written by Ombudsman employee + Letter written by Ombudsman employee to a healthcare organization or to the County Governor + complaint written by patients or next of kin	2	2
Total number of complaints	250	221

*Ethical considerations*

The data used in our study were not produced for research purposes. Extra precautions were therefore taken while ethical considerations were considered. To facilitate the acquisition of necessary data and ensure adherence to ethical guidelines, data protection laws, and protocols, applications were submitted to several bodies.

An application (reference no. 257638) was submitted to the Regional Committee for Medical and Health Research Ethics (REC). The REC responded that they could not make decisions on data from the Ombudsman, as Ombudsman employees are not healthcare professionals and therefore fall outside the scope of the Health Research Act.

A key ethical concern was the difficulty of obtaining consent due to the time lapse since the participants’ contact with the Ombudsman. To address this, an application (reference no. 638208) was sent to the Norwegian Agency for Shared Services in Education and Research (SIKT). SIKT advised us to complete a data protection impact assessment to assess the risks of personal data breaches. Based on this assessment, SIKT granted approval for the processing of personal data in the project, despite the fact that not all participants could be notified about the project.

Subsequently, the Norwegian Directorate of Health, the agency responsible for our data set, granted us permission (reference no. 22/2346-4) to access data typically protected by confidentiality under the Public Administration Act [23], waiving confidentiality requirements specifically for research purposes. An additional application was submitted to the ethics committee at the Faculty of Health and Sport Sciences at the University of Agder which also approved the study.

After receiving these approvals, an Ombudsman employee securely uploaded the data onto the University of Oslo’s TSD (The Secure Data Service) platform, which safeguards sensitive research data [24].

The data protection impact assessment helped us understand that the benefits of the study to patients and the public outweigh the potential harm from using complaints without consent. To minimize harm, the 72 individuals identified by name, email, or home address in the complaints were notified about the study via letter or email, giving them a chance to contact AAE with questions. While a few inquired, only two opted out. The remaining 149 individuals were not identified in the data and were not notified.

The data were thoroughly anonymized to protect participant privacy, with personally identifiable information removed or transformed. The original text was in Norwegian. AAE translated quotes from the documents to English to illustrate our findings.

### Analysis

AAE extracted key details from the complaints, such as the participants’ gender and healthcare services mentioned. The information was organized in an Excel spreadsheet to provide background information on the complainants and their complaints.

The data were uploaded to NVIVO 2020 for reflexive thematic analysis, following Braun and Clarke [25] six phases. In phase 1, AAE familiarized herself with patient and next-of-kin issues in primary care, treating the data as constructionist conceptualizations. This approach views language as dynamic and symbolic, where meaning is actively constructed through how we talk and write [25]. Specific texts selected by AAE were read and discussed by all authors, focusing on if it was patients, next of kin or Ombudman employees who had written the texts, the issues described and how they were presented by the author. We found that texts written by patients and next of kin were more subjective, offering deeper insight into their personal experiences with negative aspects of healthcare. In contrast, notes written by Ombudsman employees were more objective and bureaucratic, presenting the real issues with healthcare services more clearly.

The data underwent systematic coding (phase 2), aiming to preserve data closeness and encompass multiple meanings, using both semantic (explicit) codes and latent (implicit) codes. In phase 3, AAE developed seven initial themes reflecting patterns in the data and began drafting the findings, during which the themes were further refined (phase 4). All authors met several times to discuss the initial themes. Much of the discussion centred on AAE’s interpretation of the data, with TEF and IBL drawing on their prior experience in qualitative text analysis to explore alternative interpretations. AAE’s clinical background as a nurse often led her to specific conclusions, while TEF and IBL encouraged a broader perspective by challenging her to consider different angels.

Themes were then reviewed by all the authors to determine if they would tell a clear story of the analysis, and after further analytical work, the initial themes were reduced to five themes (phase 5). In a workshop with a diverse reference group, AAE presented narratives representing the themes without disclosing the specific theme names. The group members individually shared their thoughts, insights, and interpretations. This process refined and expanded the themes to capture diverse realties and perspectives.

**Fig. 1:**
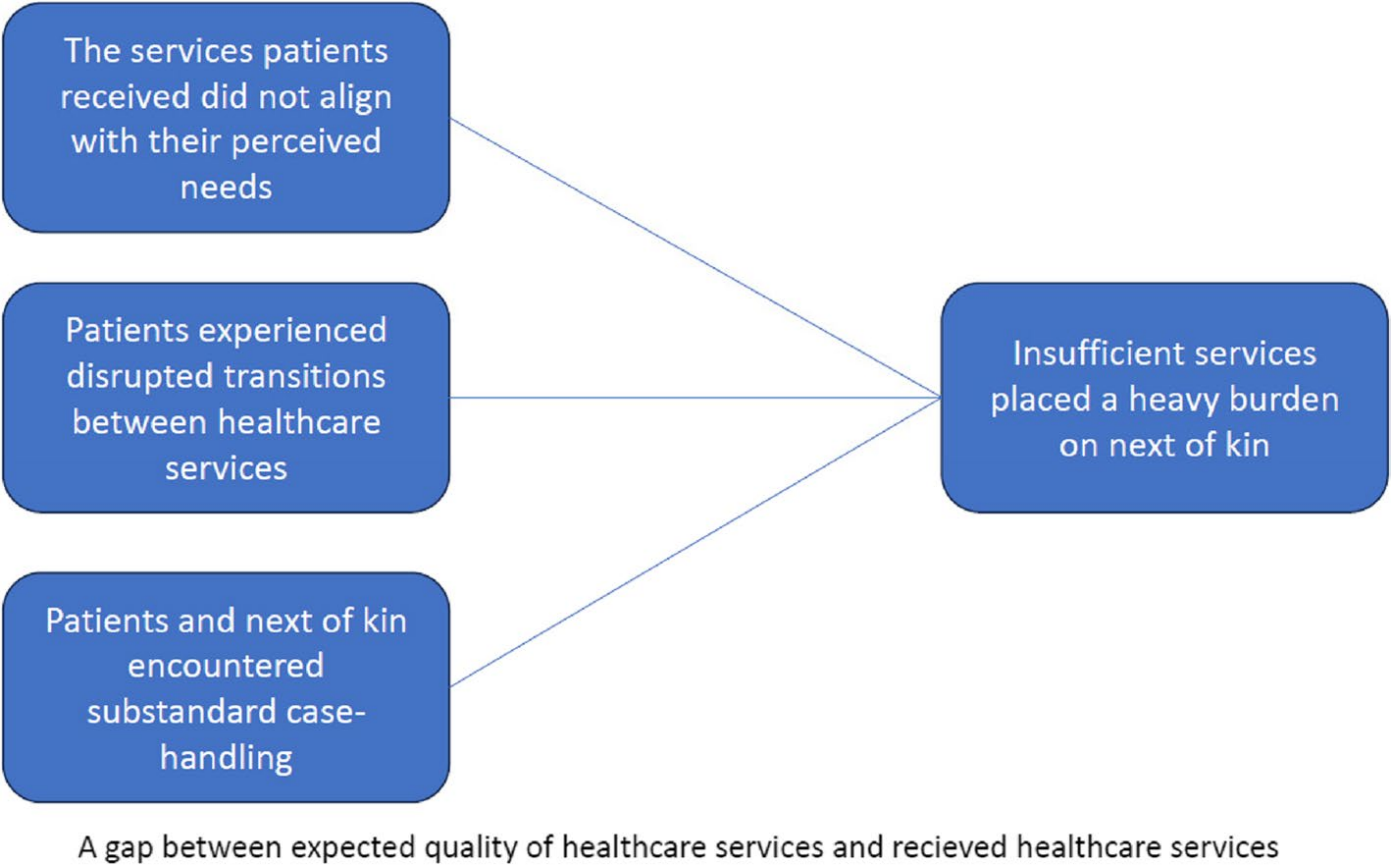
A gap between expected quality of healthcare services and received healthcare services

Discussions with TEF and IBL ensured theme consistency and alignment with the study’s aim. A thematic map (Fig. 1) and four final themes were then generated (phase 6). Three themes refer to a gap between the expected quality of healthcare services and the services received, whereas the fourth theme describes the burden on patients’ next of kin due to insufficient services. The four themes are as follows: 1) the services patients received did not align with their perceived needs; 2) patients experienced disrupted transitions between healthcare services; 3) patients and next of kin encountered substandard case handling; and 4) insufficient services placed a heavy burden on next of kin.

Table 2 shows an example of the text, codes, initial themes and final themes from the analytical process.

**Table 2:** The analytical process

**Text**	**Code**	**Initial themes**	**Themes**
The client shows up at the reception. She has been seeing her current general practitioner for four years and feels that she is not being taken seriously or getting the right referral. She is struggling with pain in her body and thinks it might be a rheumatic disease or fibromyalgia.	feels symptoms are not acknowledged by healthcare professional	Experiencing inappropriate care and bad treatment	The services patients received did not align with their perceived needs

### Reflexivity

Reflexivity, i.e., recognizing the researchers’ influence in the analytical process, is central to reflexive thematic analysis [25]. The research team consists of three members. AAE is a female nurse specializing in patient education with a keen interest in patient experience research. TEF is male and has extensive expertise in health science and qualitative research. IBL is female and has a comprehensive background in psychosocial health and psychiatric nursing, with expertise in qualitative research, particularly in relation to community health. The reference group represented a patient (male), a next of kin (female), two employees in primary care (females) and two Ombudsman employees (females). All those involved in the analytical process contributed their experiences with healthcare services both as clinicians and users, along with their academic background and creativity, to the analytical process. This means that our backgrounds helped us understand and interpret the written documents in a manner that others without backgrounds from healthcare might have understood and interpreted differently.

## Results

### Characteristics of the study sample

Not all the documents included the age of the patient and/or the complainant, and to protect their identities, no more information than what was included in the text was extracted from the Ombudsman database. Table 3 outlines the study sample’s characteristics, including primary care services and complainants. When multiple services were mentioned, the most prominent service was categorized. Nearly half of the complaints pertained to services used by the general population (short-term healthcare services), encompassing GPs, dentists, physiotherapists, EMCs and health care centres. Moreover, nearly the same amount of care is related to long-term care, including nursing homes, sheltered homes, emergency care beds, short-term relief stays, home care, mental healthcare and UPA. While most complaints cited specific healthcare services, not all addressed individual healthcare professionals. A small number of complaints concerned case handling and administration. Patients predominantly filed complaints about short-term services, whereas long-term services garnered more complaints from next of kin, mostly females.

**Table 3:** The characteristics of the study sample

**Primary Care Services**	**Patients**			**Next-of-kin**			**Other person**	**Total**
	**Female**	**Male**	**N/A**	**Female**	**Male**	**N/A**		**n**
General Practitioner (GP) or intermediary GP	35	27	2	14	4		1	83
Dentist		1						1
Physiotherapist	2	1						3
Emergency Medical Centres (EMC)	2	5		3	1			11
Health Care Centres/Health Care Centre services in schools	1			1				2
Nursing homes	1	3		16	9	1	1	31
Sheltered homes	1	1		3	1	1		7
Emergency care beds/Short-term care at nursing homes	2	3		9	3			17
Short-term relief stays				2	2			4
Homecare	4	3		10	4		2	23
Mental health care/substance abuse services	1	1		2	1			5
User-controlled personal assistance (UPA)	2			4	4			10
Administration/Case handling	1	4		8	2	1	2	18
Unclear what services complaint is about	4	2						6
**Total amount of complaints**	**56**	**51**	**2**	**72**	**31**	**3**	**6**	**221**

### Presentation of the results

Themes describing patients’ and next-of-kin problems with primary services developed through thematic analysis are presented below.

### The services patients received did not align with their perceived needs

Complaints forwarded to the Ombudsman indicate that patients and next of kin experienced that the services patients received did not align with their perceived needs. Some of these experiences related to access to services while others related to the way the service was provided or the actual content of the service.

A few complainants reported that it was difficult to schedule an appointment with the GP or to access rehabilitation services as well as delays in receiving EMC care. Patients receiving home care services reported suboptimal services and difficulties adjusting services when their health worsened. One patient expressed this concern as follows:

*It is incomprehensible that I now have more tasks, requirements and needs than I had when I was younger, but I get significantly less help.* (Case no. 75)

An Ombudsman employee wrote the following in a complaint to one municipality when the same patient applied for UPA and was allocated homecare services instead:

*Home care nurses visit at a fixed schedule during the night. The client says that she rarely needs to visit the bathroom when the nurse comes, and most of the time, she is woken up for nothing. This is very disturbing for her sleep, and it affects her day negatively.* (Case no. 75)

Other patients complained to the Ombudsman about being provided with homecare services when they applied for around-the-clock healthcare assistance. Some relatives complained about elderly parents being placed in nursing homes without their consent. Others faced challenges accessing both short- and long-term care in nursing homes, with long-term care sometimes assigned to facilities other than the patient’s preferred choices.

Patients and next of kin complained that healthcare professionals did not take their concerns seriously, especially during emergencies or health deterioration. They cited inadequate examinations, poor follow-up of test results, and missed diagnoses, which sometimes led to severe health complications and suffering. One Ombudsman employee noted the following after speaking to a patient who had breast cancer 20 years ago:

*The client had consultations with her GP several times in these last months because of recurring symptoms, but the doctor said that it was muscular and did not want to send a referral. After the client insisted, she was sent to a CT/MR, which indicated that the cancer was back and had spread.* (Case no. 6)

The next-of-kin to patients receiving home care or staying in emergency care beds or nursing homes complained about insufficient and delayed care. When these problems are raised, healthcare professionals often excuse the unmet needs by stating that healthcare cannot be forced on individuals. Conversely, some complained about receiving assistance for tasks they could handle themselves while lacking support for essential tasks with which they needed help.

The next of kin expressed concerns that when their family members’ health declined, medical treatment was withheld, and they assumed that this was an intentional decision to hasten their relatives’ demise. Patients and next of kin also expressed concerns about the absence of physical and other stimulating activities, especially for individuals living alone or in nursing homes, rendering them passive and lacking in engagement.

### Patients experienced disrupted transitions between healthcare services

The complaints indicate problems in the transitions between different kinds of healthcare services, leading to fragmented care, delays, and confusion because of patients’ lack of information.

Patients raised concerns about their GPs sharing information with other authorities without notification or discussion during subsequent visits. For example, some patients had their licences revoked due to declining health or were reported to child welfare for substance abuse. Patients claimed that their GPs did not provide essential information to welfare services, hindering their access to welfare benefits. Patients also encountered issues with referrals written by their GPs for specialized healthcare services. According to patients, some referrals lacked sufficient details about their health, resulting in delays or denials of specialized care access. One Ombudsman employee noted the following:

*The client called and is upset about the waiting time for an orthopaedic operation, and he is questioning what kind of information lays behind this prioritization. The client says he has been referred to the hospital by his GP for about two years ago because of hip pain. Eventually it appears that the client has several challenges, with his foot, ancle, knee, and the patient wonders if his GP has written an inadequate referral.* (Case no. 22)

Patients frequently reported issues encountered during transitions following hospitalization. Some were surprised to be offered home care instead of nursing home placement, despite receiving the impression that they required nursing home care from hospital healthcare professionals. Others complained of a lack of information, including contradictory details, as they prepared for discharge, especially when substantial care was still needed. Some relatives reported issues when inexperienced staff members were assigned to provide home healthcare. One relative described their experience:

*My mother cannot speak because of the ventilator. She is physically weak due to the disease and thus dependent on help for everything. She feels that the training is sometimes not good enough. She must deal with personnel who are not fully trained and do not know her well enough. This means that she must expend effort in explaining and teaching, which she experiences as exhausting and frustrating.* (Case no. 133)

### Patients and next of kin encountered substandard case handling

The complaints also indicate that patients and next of kin encountered several issues with the processing of applications for primary care services.

Complaints suggest delays in receiving responses to healthcare service applications. Frequently, applicants received negative verbal responses but had to wait for the written version, making it challenging to appeal these decisions. Some patients with access to multiple services needed a coordinator to establish an individual healthcare plan, but they complained that such a plan was never created or implemented. Others expressed dissatisfaction with the lack of information about their healthcare rights and the application process. One relative mentioned this issue in a complaint letter:

*On behalf of my uncle, I would like a meeting with the application office. As I see it, my uncle needs more information about the application process and decisions that were taken, he needs to understand the regulations, what kind of healthcare services he is entitled to, and how to effectuate these. On the other hand, healthcare service providers need more information to be able to find out more about his health and needs.* (Case no. 72)

Other patients highlighted concerns about receiving inaccurate information regarding service costs, causing financial difficulties, especially for those relying solely on welfare benefits. Some patients received access to healthcare services but lacked guidance on initiation, whereas others experienced reductions in services despite no changes in their situations.

### Insufficient services placed a heavy burden on next of kin

Individuals with children or elderly relatives with substantial healthcare needs reported that they carry an enormous part of the burden of providing care for their family members.

Parents of children with significant healthcare requirements complained that their children needed constant care, leaving little time to attend to their own needs or those of their other children. Relatives of elderly individuals living at home with debilitating diseases mentioned that caregiving negatively impacted their own physical and mental health. They experienced physical exhaustion from providing care and anxiety about leaving their ill family members alone for extended periods. This situation has also hindered their ability to work or maintain a social life. One son expressed concern about his mother’s well-being as the caregiver of his father:

*I am now worried that it will be too much for her. She was previously very active and used to regularly go out for walks. She doesn’t go out for walks anymore. We try to encourage her to go outside, but it is difficult for her because of his condition and all the extra work. She is starting to give up and is about to break down. She can’t take it anymore and wants to leave.* (Case no. 24)

The next of kin reported that despite communicating their caregiving limits and requirements to healthcare providers, they often had to provide more care than they were comfortable with due to insufficient healthcare services. One daughter expressed her distress when she had to shorten her holiday because services did not adequately address her mother’s needs:

*I had to cancel my holiday once again. While I was still on the ferry boat, my mother called and complained about the pain.* (Case no. 211)

Some relatives could no longer tolerate the situation. One wife sought assistance from the Ombudsman when her husband was denied care at a nursing home:

*After a telephone conversation with you, I received two different doctor certificates confirming my situation. At my age, it is impossible for me to have my husband in home care. An even more important point, with our economy, we cannot pay private nursing home care for much longer. I hope you can help me.* (Case no. 215)

Other relatives were willing to provide care themselves but encountered challenges in securing respite from their caregiving responsibilities. The number of relief days offered decreased each calendar year, making it difficult for them to prioritize their own well-being while providing care.

### Integrating the findings to a patient-centred framework

The four themes identified in this study illustrate various negative experiences in primary care. To provide a structured understanding of these findings, they were mapped onto the Patient-Centred Depiction of Quality Framework [26], which emphasizes quality domains from the patients’ perspective. This framework offers a lens through which the findings can be contextualized within the broader concept of patient-centred care. Table 4 is constructed using data from the participants in this study, categorizing them under the framework’s domains and providing practical advice to healthcare providers and professionals, derived from the findings.

**Table 4:** Using the patient-centred depiction of quality framework to conceptualize findings

Our Findings-Learning Opportunities	Patient-Centred Depiction of Quality Framework Domains	Giving Our Findings a Patient or Next of Kin Voice – Advice to Healthcare Providers and Healthcare Professionals
• Difficulties with access to services-GP, rehabilitation and EMC care • Difficulties with access to the right level of care for patients dependent on long-term healthcare services • Patients were placed in nursing homes without next of kin consent • Next-of-kin observed that medical treatment was withheld when the patient’s health declined	Provide me with the right care	• Provide easy access to short-term healthcare services such as GP, rehabilitation, and EMC care • Ensure access to the appropriate level of care when I need long-term healthcare services • Do not place me in a nursing home without my consent or that of my next of kin • Inform me and my family about medical decisions concerning my care, especially when my health declines
• Not being taken seriously by healthcare professionals • Inadequate examinations • Poor follow-up on test results • Missed diagnoses	Keep me safe	• Take me and my healthcare problems seriously • Provide me with adequate examinations • Follow up my test results within a reasonable timeframe • Give me the right diagnosis
• Insufficient or delayed care in homecare, emergency care beds and nursing homes • Patients received assistance for tasks they could handle while lacking support for essential tasks which they needed help with • Patients living in nursing homes were not physically active and they did not take part in stimulating activities • Parents of children with significant healthcare requirements had no time to care for their other children or themselves • Relatives of elderly living at home with significant healthcare needs experienced a negative impact of providing informal care	Help me stay well	• Provide me with sufficient and timely care when I need long-term healthcare services • Assist me with tasks I need help with, but let me do the tasks I can handle myself • Keep me physically active and help me engage in meaningful activities while receiving long-term healthcare services • Provide me, as a parent to a child with significant healthcare needs, respite services so I can take care of my other children and myself • Provide me, as a relative of an elderly person living at home with significant healthcare needs, respite services so that I can take care of my own health
• GPs did not provide essential information to welfare services hindering access to benefits • Some referrals for specialized healthcare services lacked sufficient health details resulting in delays or denials of access to care • Patients were given wrong information about the hospital-home transition • Patients were met by inexperienced staff at their home after hospital transitions • Patients experienced delays in receiving responses to healthcare service applications • Patients received negative responses verbally • Patients with access to multiple services did not get an individual healthcare plan • Lack of information about healthcare rights • Lack of information about application process • Patients received inaccurate information regarding service costs • Patients received no guidance on service initiation upon access	Help me navigate my care	• To my GP: Provide essential information to other welfare services so that I can get access to welfare benefits • To my GP: Write detailed referrals that clearly explain my health issues when I need specialized care • Plan my transition from hospital care to home carefully and promptly, and provide me and my family with accurate information about the process • Provide me with homecare staff who have the right level of competence when I am transferred from hospital to my home • Respond to my applications in writing within a reasonable timeframe • Provide me with an individual healthcare plan if I have access to multiple services • Give me information about primary care services • Give me information about the application process for primary care services • Provide me with accurate information about the cost of services • Provide me with information about how services are initiated
• GPs shared information with other authorities without informing the patients • Patients placed in nursing homes other than their preferred choices • Healthcare professionals excused unmet healthcare needs by stating that healthcare cannot be forced on individuals • Experienced reduction in services despite no changes in their health situation • Next of kin had to provide more informal care than they were comfortable with	Treat me with respect	• To my GP -Do not share information about me without informing me • Allow me to have a say in which nursing home I am placed in • Explain to my next of kin the reasons behind certain decisions about my care and involve them in decisions regarding my care • Do not reduce my services unless there has been a change in my health situation • Allow me, as a relative of an elderly person living at home with significant healthcare needs, to have a say regarding the informal care I can provide

## Discussion

This study provides an understanding of negative experiences with primary care in Norway, as expressed in complaints to the Ombudsman. Our findings show that patients have issues with both short- and long-term services, highlighting issues that align with earlier research, such as healthcare access, poor coordination, and inadequate treatment [13]. However, our study extends the literature on complaints by revealing a gap between the quality of expected and received healthcare services, which directly impacts patients while also placing a significant burden on next of kin. These findings raise concerns about the perceived quality of primary care services, which requires further discussion.

### A gap between the quality of expected and received healthcare services

The findings from this study show that patients did not receive healthcare services of the quality that they expected. Patients in need of short-term healthcare services could identify poor-quality care. These findings are similar to findings from existing research on complaints about GP services, indicating that patients commonly express concerns about safety issues, encompassing errors, substandard care and safety incidents [27]. Although complaints about quality issues are expected to arise when patient expectations are not met, these should still be taken seriously since patient feedback can uncover legitimate mistakes [27]. To safeguard patient well-being, it is imperative that GPs employ effective methods of quality assurance.

Similarly, patients requiring long-term healthcare services reported that the services provided did not meet their needs adequately. Some patients who applied for UPA received home care services, whereas others who applied for long-term care in nursing homes received home care with occasional respite. Thus, our findings suggest that patients may receive services that are the next best alternative compared with their initial preferences or requests. These findings are consistent with the observations outlined in the 2019 Ombudsman report, which highlights a significant gap between the healthcare level that municipalities determine patients require and the level that healthcare patients perceive they need [21].

These complaints might be a result of a mismatch in patient expectations; however, they could also serve as indicators of missed care. Missed care can be defined as any element of necessary patient care that is either partially or entirely neglected or postponed [28]. Although nurses typically identify missed care when facing time constraints and the need for prioritization, a recent study reported that patients are also capable of recognizing instances of missed care [29].

Our study highlights that next of kin possess the ability to identify instances of missed care and, on occasion, proactively communicate their observations to healthcare professionals. However, some participants in this study reported that healthcare professionals justified instances of missed care by asserting a hesitancy to compel patients to accept services they did not desire. Ethical conflicts can arise when healthcare professionals respect patients’ autonomy [30]. Healthcare professionals demonstrate a willingness to employ coercion when patients decline healthcare, only when the absence of such coercion may cause direct harm to the patient or put the patient at a safe risk [30]. Healthcare professionals refraining from providing care to respect patients’ wishes might be seen by next-of-kin as a lapse in healthcare service quality.

The findings of this study highlight the disparity between expected and received healthcare services in terms of inadequate application handling and challenges in transitioning between various healthcare services. Earlier research has indicated that patients’ needs and expectations are variable and can change on the basis of the care received and patients’ understanding of outcomes at both the healthcare organization level and the individual level [31]. Thus, both the organization of healthcare and the way it is delivered by healthcare professionals impact patients’ expectations of care. Additionally, patients' expectations and interpretations of their care experiences are influenced by broader societal, community, and family contexts [31].

Given the many factors influencing patient expectations, it is worth questioning whether the healthcare system can realistically meet all of these expectations, particularly in contexts where healthcare resources are limited. Nonetheless, the Patient and User Rights Act [6] grants patients the right to safe, good-quality healthcare and the UN’s standard of health (Art. 12) affirms the right to achieve the highest attainable standard of health [32]. States are obliged to ensure that the necessary resources are available [32]. Is it, however, enough for the state and healthcare providers to define “good quality” or should patients and next of kin also have a voice?

Quality in healthcare is often defined by the six domains established by the Institute of Medicine framework, which state that high-quality care should be: safe, effective, patient-centred, timely, efficient and equitable [33]. These domains are also recognized in Norwegian healthcare standards [34, 35]. While our findings align with these domains, they reveal a gap between the expected quality of care and the received quality of care, which might indicate a need for more patient-centred care approaches. Given this, a patient-centred framework that integrates these six domains could offer valuable insights for identifying learning opportunities. Consequently, we applied the Patient-Centred Depiction of Quality framework [26], presented in Table 4, to help contextualize and interpret our findings. This integration aims to guide healthcare providers and professionals in understanding the needs of patients and next of kin, helping to prioritize what matters most to patients and families, ultimately informing healthcare decisions. While the state and healthcare providers may define “good quality”, it is essential that patients and next of kin also have a voice in determining what constitutes quality care, ensuring that healthcare services reflect their needs and priorities.

### Impact on the next of kin

On the basis of these results, next of kin sometimes feel compelled to offer informal care to their relatives because the quality of healthcare services received is not as good as expected. These findings imply that when subpar healthcare is provided, next of kin may become motivated to offer informal care to their family members, even when initial willingness is lacking. This aligns with prior research showing that, owing to an aging population and limited healthcare resources, governments favour informal care over formal care [36]. This involves promoting home-based healthcare over institutions and nudging next of kin to provide informal care [36]. However, this approach raises several concerns.

First, if next of kin are not initially willing to offer informal care, will they provide good-quality care over time? Research shows that reluctance negatively affects caregiver, patient, and family well-being [37]. Next of kin admitting reluctance report higher depression levels and tend to provide lower-quality care, potentially even engaging in behaviours that are harmful to the patient [37]. Therefore, healthcare provision by initially unwilling next of kin may raise concerns about service quality.

Second, the effect of informal care on next of kin must also be considered. Our study shows that informal care is experienced as a heavy burden by some next of kin, resulting in negative consequences for their own health. Moreover, next of kin who are motivated to care for their family members may encounter difficulties because of reduced offers of respite services. Previous studies support our findings regarding the negative impact of the next-of-kin burden [36, 38]. The duration of caregiving and patients’ dependency level significantly contribute to caregiver burden [36]; thus, social support [36] and respite services are crucial incentives for next of kin to continue providing informal care [37]. However, our study highlights a trend of decreasing support for informal caregivers, which poses challenges to their sustained role and compromises their health and safety.

### Strengths and limitations of the study

This study provides a detailed account of patient and next-of-kin complaints regarding primary care services in Norway. However, this study has limitations. First, the study’s sample is limited to one year. Second, the experiences are solely from the viewpoint of patients or their next of kin and may not encompass the full healthcare context. Third, most complaints were documented by Ombudsman employees, capturing their interpretations rather than the direct experiences of patients and relatives. Nevertheless, some cases included multiple documents, offering rich qualitative data for thematic analysis. However, other research methods, such as interviews, would have given the researchers the opportunity to ask follow-up questions and obtain an even more detailed account of the participants’ experience.

While patient experience measures offer valuable insights for policymakers focusing on quality improvement initiatives, the methodologies employed to gauge these measures are pivotal in determining the utility of the findings [31]. Qualitative methods, which provide subjective measures of care quality, are instrumental in identifying potential areas for enhancement in healthcare services [31]. Conversely, quantitative methods, which are claimed to be objective, are better suited for assessing whether the level of healthcare services provided meets acceptable quality standards [31]. Considering these factors, our study highlights specific areas in healthcare where patients feel dissatisfied, offering healthcare providers a direction for potential improvements. However, it is important to note that, even though our study reveals a discrepancy between the expected and received quality of care from the patients’ perspective, it does not necessarily imply that the quality of care provided is substandard.

While this study uses qualitative methods, its large sample size allows it to contribute meaningfully to discussions about primary care in Norway. This study can offer insights on what patients and next of kin need from primary care providers. However, given that patient expectations are influenced by specific contexts, these findings should be applied with consideration to the unique circumstances of each healthcare setting.

### Implications for practice, research, and policy

This study provides an understanding of negative experiences with primary care in Norway, offering a deeper understanding of patient and next-of-kin expectations from healthcare service providers and professionals. These findings not only are relevant to the Norwegian context but can also inform healthcare practices in other countries facing similar challenges. Healthcare professionals and students in various countries can use this information for self-reflection, helping to improve their practices and thereby enhancing the quality of care.

Further research should establish whether the quality of primary services is at an acceptable level. Additional research is needed to determine the quality of services provided by informal caregivers and the impact that informal caregiving has on the health of next of kin. Crucially, healthcare policies should focus on how more support can be given to next of kin providing informal care.

Whether identified by healthcare professionals, patients, or next of kin, instances of missed care demand serious attention from healthcare organizations.

Healthcare professionals can play a pivotal role in shaping patient expectations by engaging in discussions about health outcomes with patients. This interaction becomes particularly crucial in instances where health outcomes deviate from what patients or next of kin anticipate. By providing clear and empathetic communication, healthcare professionals can help adjust patient expectations and facilitate a better understanding of their health situations. At the same time, healthcare professionals should focus on involving patients and next of kin in decision-making and providing patient-centred care.

Policies should implement better quality measures for municipalities, ensuring that patient feedback is effectively utilized for continuous quality improvement.

## Conclusions

This study highlights issues in Norwegian primary care services, revealing a gap between the quality of expected and received healthcare, impacting patients, and nudging next of kin to take on informal caregiver roles. This raises several concerns about the quality of care provided by both primary care professionals and informal caregivers, as well as the health implications for the caregivers themselves. A greater focus on patient-centred care could better meet patient needs, improve resource efficiency and alleviate some of the burden on the next of kin.

## Supplementary Information

Supplementary Material 1.

## Data Availability

To protect the anonymity of the participants and others mentioned in the dataset, the dataset cannot be made available to the public.

## Abbreviations

Ombudsman: Health and Social Services Ombudsman
EDQ: Explorative descriptive qualitative
COREQ: Criteria for Reporting Qualitative Studies
GP: General practitioner
EMC: Emergency medical centres
UPA: User-controlled personal assistance
REC: Regional Committees for Medical and Health Research Ethics
SIKT: Norwegian Agency for Shared Services in Education and Research
TSD: The Secure Data Service

## References

[1] Hanson K, Brikci N, Erlangga D, Alebachew A, De Allegri M, Balabanova D, et al. The Lancet Global Health Commission on financing primary health care: putting people at the centre. The Lancet Global Health. 2022;10(5):e715-e72.10.1016/S2214-109X(22)00005-5 10.1016/S2214-109X(22)00005-5 PMC9005653 35390342

[2] Saunes IS, Karanikolos M, Sagan A. Norway: Health System Review. WHO Regional Office for Europe, Denmark: world health organization; 2020. 32863241

[3] Dahlborg E, Tengelin E, Aasen E, Strunck J, Boman Å, Ottesen AM, et al. The struggle between welfare state models and prevailing healthcare policy in Scandinavian healthcare legislative documents. International journal of health governance. 2021;26(1):51-64.10.1108/IJHG-04-2020-0041

[4] Norwegian Ministry of Health and Care Services. The primary health and care services of tomorrow - localised and integrated. Meld. St. 26 (2014-2015) Report to the Storting (white paper) Summary. 2015.

[5] Solberg CT, Tranvåg EJ, Magelssen M. Attitudes towards priority setting in the Norwegian health care system: a general population survey. BMC health services research. 2022;22(1):444.10.1186/s12913-022-07806-9 10.1186/s12913-022-07806-9 PMC8980508 35382816

[6] Lov om pasient- og brukerrettigheter (pasient- og brukkerrettighetsloven) [The Patient and User Rights Act]LOV-1999-07-02-63. , (1999).

[7] Lov om kommunale helse- og omsorgstjenester m.m. (helse- og omsorgstjenesteloven) [Act relating to municipal health and care services] LOV-2011-06-24-30, (Last update 01.01.2012).

[8] McCance T, McCormack B. Developing healthful cultures through the development of person-centred practice. International journal of orthopaedic and trauma nursing. 2023;51:101055-.10.1016/j.ijotn.2023.101055 10.1016/j.ijotn.2023.101055 37857103

[9] Ricci-Cabello I, Pons-Vigués M, Berenguera A, Pujol-Ribera E, Slight SP, Valderas JM. Patients’ perceptions and experiences of patient safety in primary care in England. Family practice. 2016;33(5):535–42. 10.1093/fampra/cmw046. 27312563 10.1093/fampra/cmw046

[10] Van Dael J, Reader TW, Gillespie A, Neves AL, Darzi A, Mayer EK. Learning from complaints in healthcare: A realist review of academic literature, policy evidence and front-line insights. BMJ Quality & Safety. 2020;29(8):684-95.10.1136/bmjqs-2019-009704 10.1136/bmjqs-2019-009704 PMC7398301 32019824

[11] Reader TW, Gillespie A, Roberts J. Patient complaints in healthcare systems: A systematic review and coding taxonomy. BMJ Qual Saf. 2014;23(8):678-89.10.1136/bmjqs-2013-002437 10.1136/bmjqs-2013-002437 PMC4112446 24876289

[12] Harrison R, Walton M, Healy J, Smith-Merry J, Hobbs C. Patient complaints about hospital services: Applying a complaint taxonomy to analyse and respond to complaints. International Journal for Quality in Health Care. 2016;28(2):240-5.10.1093/intqhc/mzw003 10.1093/intqhc/mzw003 26826722

[13] Eriksen AA, Fegran L, Fredwall TE, Larsen IB. Patients' negative experiences with health care settings brought to light by formal complaints: A qualitative metasynthesis. Journal of Clinical Nursing. 2023;32(17-18):5816-35.10.1111/jocn.16704 10.1111/jocn.16704 36975841

[14] Jones J, Bion J, Brown C, Willars J, Brookes O, Tarrant C, et al. Reflection in practice: How can patient experience feedback trigger staff reflection in hospital acute care settings? Health Expectations. 2020;23(2):396-404.10.1111/hex.13010 10.1111/hex.13010 PMC7104653 31858677

[15] Charon R. At the membranes of care: stories in narrative medicine. Academic medicine. 2012;87(3):342-7. 10.1097/ACM.0b013e3182446fbb 10.1097/ACM.0b013e3182446fbb PMC3292868 22373630

[16] Hunter D, McCallum J, Howes D. Defining exploratory-descriptive qualitative (EDQ) research and considering its application to healthcare. Journal of Nursing and Health Care. 2019;4(1).http://eprints.gla.ac.uk/180272/

[17] Tong A, Sainsbury P, Craig J. Consolidated criteria for reporting qualitative research (COREQ): a 32-item checklist for interviews and focus groups. International journal for quality in health care. 2007;19(6):349-57.10.1093/intqhc/mzm042 10.1093/intqhc/mzm042 17872937

[18] Bowen GA. Document analysis as a qualitative research method. Qualitative research journal. 2009;9(2):27.https://doi.org./10.3316/QRJ0902027

[19] Neumann CB, Gundersen T. Care parading as service: Negotiating recognition and equality in user‐controlled personal assistance. Gender, Work & Organization. 2019;26(7):948-61.10.1111/gwao.12297

[20] The Health and Social Services Ombudsman. Health and Social Services Ombudsman Oslo: The Directorate of e-health; 2022 [Available from: https://www.helsenorge.no/en/health-rights-in-norway/pasient--og-brukarombodet/.

[21] The Health and Social Services Ombudsman. Årsmelding 2019 pasient- og brukerombudene [Annual Report 2019 from The Health and Social Services Ombudsman] Oslo2019 [

[22] Bøe TD, Bertelsen B, Larsen IB, Topor A. A qualitative fallacy: Life trapped in interpretations and stories. Qualitative Research. 2021:14687941211041916.10.1177/14687941211041916

[23] Act relating to procedure in cases concerning the public administration (Public Administration Act), Public Administration Act(1967).

[24] Øvrelid E, Bygstad B, Thomassen G. TSD: A Research Platform for Sensitive Data. Procedia Computer Science. 2021;181:127-34.10.1016/j.procs.2021.01.112

[25] Braun V, Clarke V. Thematic analysis : A practical guide. Los Angeles, California: SAGE; 2022.

[26] Insititute for Healthcare Improvement. What Boards Must Do to Acheive Better Quality Health Care 2019 [Available from: https://www.ihi.org/insights/what-boards-must-do-achieve-better-quality-health-care.

[27] O’Dowd E, Lydon S, Madden C, O’Connor P. A systematic review of patient complaints about general practice. Family practice. 2020;37(3):297-305.10.1093/fampra/cmz082 10.1093/fampra/cmz082 31742596

[28] Kalisch BJ, Landstrom GL, Hinshaw AS. Missed nursing care: a concept analysis. Journal of advanced nursing. 2009;65(7):1509-17.10.1111/j.1365-2648.2009.05027.x 10.1111/j.1365-2648.2009.05027.x 19456994

[29] Gustafsson N, Leino-Kilpi H, Prga I, Suhonen R, Stolt M, Action–CA15208 RcC. Missed care from the patient’s perspective–a scoping review. Patient preference and adherence. 2020:383-400.10.2147/PPA.S238024 10.2147/PPA.S238024 PMC7049852 32161449

[30] Heggestad AKT, Magelssen M, Pedersen R, Gjerberg E. Ethical challenges in home-based care: A systematic literature review. Nursing ethics. 2021;28(5):628-44.10.1177/0969733020968859 10.1177/0969733020968859 33334250

[31] Larson E, Sharma J, Bohren MA, Tunçalp Ö. When the patient is the expert: measuring patient experience and satisfaction with care. Bulletin of the World Health Organization. 2019;97(8):563.10.2471/BLT.18.225201 10.2471/BLT.18.225201 PMC6653815 31384074

[32] UN. Committee on Economic SaCR. General comment no. 14 (2000), The right to the highest attainable standard of health (article 12 of the International Covenant on Economic, Social and Cultural Rights). Geneva: United Nations; 2000

[33] Agency for Healthcare Research and Quality R, MD.,. Six Domains of Healthcare Quality 2022 [updated December 2022. Available from: https://www.ahrq.gov/talkingquality/measures/six-domains.html.

[34] Norwegian Ministry of Health and Care Services. High Quality - Safe Services. Quality and Patient safety in the Health and Care Services. Meld. St. (2012-2013) Report to the Storting (white paper) Summary. 2013.

[35] Helsedirektoratet [The Norwegian Directorate of Health]. Nasjonal handlingsplan for paisentsikkerhet og kvalitetsforbedring 2019-2023 [National Action Plan for Patient Safety and Quality Improvement 2019-2023]. Oslo2019.

[36] Lindt N, van Berkel J, Mulder BC. Determinants of overburdening among informal carers: a systematic review. BMC geriatrics. 2020;20(1):1-12.10.1186/s12877-020-01708-3 10.1186/s12877-020-01708-3 PMC7448315 32847493

[37] Zarzycki M, Morrison V. Getting back or giving back: Understanding caregiver motivations and willingness to provide informal care. Health Psychology and Behavioral Medicine. 2021;9(1):636-61.10.1080/21642850.2021.1951737 10.1080/21642850.2021.1951737 PMC8281125 34345534

[38] Liu Z, Heffernan C, Tan J. Caregiver burden: A concept analysis. International journal of nursing sciences. 2020;7(4):438-45.10.1016/j.ijnss.2020.07.012 10.1016/j.ijnss.2020.07.012 PMC7644552 33195757

[39] McNutt MK, Bradford M, Drazen JM, Hanson B, Howard B, Jamieson KH, et al. Transparency in authors’ contributions and responsibilities to promote integrity in scientific publication. Proceedings of the National Academy of Sciences. 2018;115(11):2557-60.10.1073/pnas.1715374115 10.1073/pnas.1715374115 PMC5856527 29487213

